# Dairy farmworkers’ knowledge of the causes, risk factors, and clinical signs of bovine lameness

**DOI:** 10.14202/vetworld.2024.1789-1797

**Published:** 2024-08-20

**Authors:** Sibulele Matshetsheni, Ishmael Festus Jaja

**Affiliations:** 1 Department of Livestock and Pasture Science, University of Fort Hare, Alice 5700, South Africa; 2 Department of Agriculture and Animal Health, University of South Africa, Roodepoort, Johannesburg, South Africa

**Keywords:** foot rot, increased lying time, knowledge, lameness, milk yield, reduced milk yield

## Abstract

**Background and Aim:**

Observing lameness in dairy cows is the primary animal-based indicator of their overall welfare and health status. The study evaluated dairy farmworkers’ understanding of bovine lameness’s causes, risk factors, and clinical symptoms in Kwa-Zulu Natal Province, South Africa.

**Materials and Methods:**

Eleven dairy farms provided the data through questionnaires. The questionnaire consisted of five sections: The first section (A) gathered demographic data (age, gender, experience, level of study), and the second section (B) dealt with farm specifics (size, grazing method). Sections C, D, and E cover farm workers’ knowledge of lameness risk factors, clinical signs of lameness, and treatment and management of lameness, respectively.

**Results:**

About 85.5% of horses experience lameness during summer, contrasted to 15% during autumn. The seasonal results were statistically significant (p < 0.05). 93% of respondents identified a decrease in appetite as the most common sign of lameness, while 94% reported reduced milk yield and 90% increased lying time; a significant correlation was found between awareness of clinical signs and localities (p < 0.05). Age, gender, and occupation level (p < 0.05) were identified as contributing factors to the movement problem in the dairy industry, necessitating training for farm workers to enhance their knowledge.

**Conclusion:**

Although there is a satisfactory (81%) understanding of lameness by farm workers. There is still need for improvement, hence, farm worker training and retraining are essential for managing and reducing the instances of bovine lameness.

## Introduction

The consequence of lameness in dairy herds is a significant concern on a global scale for animal welfare, milk production, and farm economics [1–3]. Any foot or leg condition, whether infectious or non-infectious, affecting cow mobility, posture, and gait is termed lameness [4–7]. Claw lesions are the primary reason for lameness in dairy cows, encompassing both the non-infectious forms (white line disease and sole ulcer) and infectious ones (digital dermatitis, interdigital dermatitis, heel erosion, and interdigital phlegmon [8–11]). The impairment of a cow’s mobility due to lameness triggers various adverse effects, resulting in a decrease in milk production (about 20% less), hindered reproduction, insufficient weight gain, and, occasionally, the need for culling [12–15].

The detection of lameness in dairy cows serves as the most significant animal-based indicator of their overall well-being [6, 16]. Economically, lameness ranks as one of the major causes of loss on dairy farms [3, 17, 18].

Lameness, a significant issue alongside mastitis and infertility, negatively impacts animal welfare and limits the dairy industry [3, 19, 20]. Protracted calving intervals primarily cause losses, the costs of premature culling, decreased milk yield and quality, and other veterinary costs and treatments incurred by the dairy producer [15, 21, 22]. Claw disorders account for approximately 90% of all lameness in dairy cattle [11, 23, 24]. The factors influencing cow-level claw lesions include parity, lactation stage, body condition score, and genetics, while herd-level factors comprise the housing environment, management, and nutrition [7, 25–28].

Lameness in dairy cows ranks second only to mastitis for decreasing herd productivity. The cost of treating a case of lameness is dwarfed by the overall expense [6, 29, 30]. Depending on the farm, location, and year of study, the annual incidence ranges between 4 and 55 cases per 100 cows per year [31]. Reducing milk yield and increasing the probability of culling are consequences of lameness in dairy cows [15, 22, 32]. Consequently, the total cost of a case of lameness is far greater than the cost of treatment alone [6, 29, 30].

Clinical infectious lameness symptoms include fever, anorexia, decreased milk yield, pain, swelling, and paralysis [4, 31–34]. The prevalence of lameness in high-producing Holstein cows is poorly studied [24, 34, 35]. The study was designed to ascertain dairy farmer workers’ understanding of lameness prevalence and risk factors in KwaZulu Natal Province, South Africa.

## Materials and Methods

### Ethical approval and Informed consent

An ethical clearance certificate (REC-270710-028-RA Level 1) with project number JAJ011SDINO1 was obtained from the University of Fort Hare Research and Ethics Committee before the start of the data collection process. Farmers were informed about the study objective, inclusion criteria, and methodology during the visit before data collection.

### Study period and location

This study was conducted from November 2022 to March 2023. Elevem commercial dairy farms in Mooi River, Kwazulu Natal Province, South Africa were included in the study. These districts are uMgungundlovu District Municipality, uMngeni District Municipality, and uMpofana District Municipality. However, Mooi River is a small town in KwaZulu-Natal, South Africa.

### Study population

The first point of contact was visiting the dairies within the Mooi River, South Africa, to seek permission to conduct a study. Twenty five farms were chosen for the study, however, only 11 farms agreed to participate and were included in the final survey. Each farm targeted approximately 30 dairy workers, including managers, supervisors, general workers, and bulk tank workers. However, depending on farm size, each dairy farm had only 4–10 workers per milking session. As a result, 155 out of possible 160 respondents (10 from 11 dairy farms) correctly responded to the survey.

### Data collection

Prior to data collection, a questionnaire survey and an online version were developed, validated, and piloted. The questionnaire was matched with similar studies conducted elsewhere [36]. Five dairy workers and two students also validated the survey. The questionnaire was divided into five sections: The first (Section A) of which contained demographic information (age, gender, experience, and level of study), and the second (Section B) contained farm information such as farm size and grazing method. However, Section C discussed farm workers’ knowledge on lameness and risk factors, Section D discussed clinical signs of lameness, and Section E discussed treatment and management of lameness. As a result, the questionnaire included closed-ended and open-ended questions, and respondents were required to write the correct answer. Furthermore, the questionnaire was written in English. However, to make it easier for respondents, it was translated to isizulus, the respondents’ language during data collection. In addition, from November 2022 to March 2023, five trained enumerators distributed the questionnaire to farm managers or owners.

### Statistical analysis

The collected data were coded and entered into Microsoft Office Excel 2016 (Microsoft, Washington, USA). The IBM Statistical Package for the Social Sciences Version 26 (IBM Corp., NY, USA) was used to calculate the frequency and percentages of the demographic variables. A total of 42 questions and statements were used to assess the respondents’ knowledge of risk factors, clinical signs, treatment, prevention, and management of lameness. The Chi-square test was used to establish statistical relationships between variables. The results were significant at p ≤ 0.05.

## Results

### Demographic information

About 63.9% of respondents were males than females (36.1%). Most respondents were between 31 and 40 years of age (40%) compared to other age groups (Table-1). Sixty-nine percent of respondents were black. 55.5% were single, 32.9% were married, 10.3% were divorced, and 1.3% were widowed. Most of the respondents attained tertiary education (37.4%), and most respondents were general workers (40.6%). Furthermore, 39.4% of respondents had 2–4 years of experience in dairy (Table-1). Herringbone (36.7%) and rotary (37.4%) had the highest frequencies than parallel (28.9%) milking parlor. Jersey (16.8%), Friesland (7.7%), and Crossbreed (3.2%) were the most dominant breeds in these dairies, with the significance for these breeds being p < 0.05. Furthermore, herd size was significant (p < 0.05) (Table-2).

**Table-1:** Demographic information (n = 155).

Variables	Category	Frequency	Percentage
Gender	Male	99	63.9
	Female	54	36.1
Age	0–20	9	5.8
	21–30	56	36.1
	31–40	64	41.3
	Above 40	25	16.1
Ethnic	Black	107	69
	Colored	19	12.3
	White	29	18.7
Marital status	Single	86	55.5
	Married	51	32.9
	Divorced	16	10.3
	Widowed	2	1.3
Education level	No formal education	18	11.6
	<Grade 12	23	14.8
	Grade 12	56	36.1
	Tertiary	58	37.4
Occupation	Owner	10	6.5
	Manager	22	14.2
	General worker	63	40.6
	Other	60	38.7
Experience	1–2	1	0.6
	3–4	61	39.4
	4–5	40	25.8
	Above 6	53	34.2

**Table-2:** Type of milking parlor, breeds, herd size and type of management system (n = 155).

Category	Variables	M1 (%)	M2 (%)	M3 (%)
Type of milking parlor	Rotary	26 (16.7)	23 (14.8)	8 (5.2)
	Parallel	2 (1.3)	35 (22.5)	3 (2)
	Herringbone	13 (8.4)	5 (3.2)	40 (25.8)
	Total	41 (26.5)	63 (40.6)	51 (32.9)
Breeds	Jersey	12 (7.7)	3 (2)	26 (16.8)
	Friesland	9 (5.8)	12 (7.7)	5 (3.2)
	Crossbreed	3 (2)	5 (3.2)	3 (2)
	Other	17 (10.9)	43 (27.7)	17 (10.9)
	Total	41 (26.5)	63 (40.6)	51 (32.9)
Herd size	1–100	3 (2)	2 (1.3)	2 (1.3)
	101–300	10 (6.5)	10 (6.5)	4 (2.6)
	301–600	2 (1.3)	4 (2.6)	4 (2.6)
	601 and above	33 (21.1)	57 (36.8)	24 (15.5)
	Total	48 (30.9)	73 (47.1)	34 (22)
Type of management system	Pasture based			
	Yes	41 (26.5)	61 (39.3)	50 (32.3)
	No	0	2 (1.3)	1 (0.6)
	Total	41 (26.5)	63 (40.6)	51 ( 32.9)
	Zero grazing			
	Yes	26 (16.8)	23 (14.8)	37 (23.9)
	No	15 (9.7)	40 (25.8)	14 (9)
	Total	41 (26.5)	63 (40.6)	51 (32.9)

### Association with farm locations

According to the current research findings, most respondents (81%) understood the definition of lameness (Table-3). There was no association (p > 0.05) between localities and knowledge of lameness risk factors. However, there was an association between (p = 0.01).

**Table-3:** Knowledge of lameness and association with farm locations (n = 155).

Questions	M1 (%)		M2 (%)		M3 (%)		Total (%)		Chi-square	p-value
			
	Yes	No	Yes	No	Yes	No	Yes (%)	No (%)		
Lameness is a severe welfare problem and a production limiting disease in a dairy farming	36 (23.2)	6 (3.9)	60 (38.7)	2 (1.3)	45 (29)	6 (3.9)	141 (90.9)	14 (9.1)	4.5	0.103
Untrimmed hoof is one of the main causes of lameness in dairy	31 (20)	10 (6.5)	56 (36.1)	7 (4.5)	43 (27.7)	8 (5.2)	130 (83.8)	5 (16.2)	3.2	0.197
The higher the increase in dairy rainfall, the higher the chance of lameness in dairy animal	35 (22.6)	6 (3.9)	60 (38.7)	4 (2.6)	46 (29.7)	4 (2.6)	141 (90.9)	14 (9.1)	2.2	0.338
Parity of an animal associated with lameness	32 (20.6)	9 (5.8)	52 (33.5)	11 (7.1)	40 (25.8)	11 (7.1)	124 (80)	31 (20)	0.4	0.807
Wounds on claw or the part of the limbs can cause lameness of lameness in the dairy animals	28 (18.1)	13 (8.4)	61 (39.4)	2 (1.3)	50 (32.3)	1 (0.6)	139 (89.6)	16 (10.3)	21.9	0.01
The infectious foot problem causing lameness as follows								
Foot rot	37 (23.8)	4 (2.6)	61 (39.4)	2 (1.3)	50 (32.3)	1 (0.6)	148 (95.5)	7 (4.5)	3.6	0.162
Interdigital dermatitis	31 (20)	9 (5.8)	40 (25.8)	25 (16.1)	37 (23.9)	13 (8.4)	108 (69.7)	47 (30.3)	2.7	0.256
Digital dermatitis	26 (16.8)	15 (9.7)	45 (29)	18 (11.6)	34 (21.9)	17 (10.9)	105 (67.7)	50 (32.3)	0.7	0.680
Mortellaro’s disease	26 (16.8)	15 (9.7)	43 (27.7)	20 (12.9)	32 (20.6)	19 (12.3)	101 (65.2)	54 (34.8)	0.4	0.798
Foot and mouth disease	37 (23.9)	4 (2.6)	59 (38.1)	4 (2.6)	45 (29)	6 (3.9)	141 (90.9)	14 (9.1)	1.0	0.594

*Statistically significance at p ≤ 0.05; M1=uMgungundlovu, M2=uMpofana, M3=uMngeni

### Association between farm locations and potential risk factors for lameness

The highest prevalence of lameness (85.5%) occurs in summer compared with other seasons, such as autumn (15%) [Table-4]. Furthermore, the results for seasons were significant (p < 0.05). There was an association (p < 0.05) between farm localities and potential lameness risk factors. The association with lameness was significant (p < 0.05) in the untrimmed data. The results for parity were significant (p < 0.05). Most respondents agreed that crossbred cows (53%) were more prevalent than Jersey (29%) and Friesland (18%). As shown in Figure-2, the fourth lactation cows were more susceptible (30%) to lameness than the other parity (3^rd^, 2^nd^ and 1^st^). In addition, respondents agreed that lameness was more prevalent in summer than in other parities.

**Table-4:** Association between farm locations and potential risk factors of lameness (n = 155).

Questions	M1		M2		M3		Chi- square	p-value
In which season is lameness common								
Summer	28.6		28.6		28.6			
Spring	0.2		0.2		0.2			
Winter	0.2		0.2		0.2			
Autumn	4.3		4.3		4.3		14.5	0.001

	**Yes (%)**	**No (%)**	**Yes (%)**	**No (%)**	**Yes (%)**	**No (%)**		

Untrimmed hoof is one of the factors that cause lameness	36 (23.0)	6 (3.9)	58 (37.4)	2 (1.3)	53 (34.2)	0	9.1	0.010
Lameness prevalence increased with the parity	36 (23.2)	8 (5.1)	52 (33.5)	10 (6.5)	42 (27)	7 (4.5)	0.5	0.798
In which parity is lameness common								
First lactation	27 (17.4)	14 (9)	47 (30.3)	15 (9.6)	31 (20)	21 (13.5)	3.6	0.153
Second lactation	34 (21.9)	7 (4.5)	52 (33.5)	11 (7.1)	36 (23.2)	14 (9)	2.3	0.309
Third lactation	35 (22.5)	6 (3.9)	55 (35.4)	8 (5.2)	39 (25.1)	12 (7.7)	2.6	0.279
Fourth lactation	32 (20.6)	9 (5.8)	59 (38)	4 (2.6)	46 (29.7)	5 (3.2)	6.1	0.047
Wounds of the lower limb increases the chance of lameness	33 (21.3)	8 (5.1)	60 (38.7)	3 (2)	49 (31.6)	2 (1.3)	9.0	0.01
Floor type	24 (15.5)	16 (10.3)	30 (19.4)	34 (21.9)	28 (18)	23 (14.8)	2.0	0.368
Animal breed is one of the risk factors if lameness	35 (22.5)	6 (3.9)	52 (33.5)	11 (7.1)	44 (28.4)	7 (4.5)	0.3	0.847
Which of the following breed is more affected by lameness								
Jersey	17 (10.9)		3 (2)		24 (15.5)			
Crossbreed	19 (12.3)		45 (29)		20 (12.9)			
Friesland	4 (2.6)		16 (10.3)		7 (4.5)		33.3	0.01

*Statistically significant at p ≤ 0.05, M1=uMgungundlovu, M2=uMpofana, M3=uMngeni

### Knowledge of clinical signs, treatment, prevention, and management of lameness

The results of this study indicate that a decrease in appetite (93%) is a clinical sign of lameness (Table-5). Nevertheless, the results also demonstrate a significant (p < 0.05) correlation between knowledge of clinical signs of lameness and localities. Other clinical signs included decreased milk yield (93%), which was statistically significant (p < 0.05). The data presented in Figure-3 show that most respondents were aware of the clinical signs of lameness. This study found that the respondents in all three localities recognized the clinical signs of lameness. In addition, fewer respondents were unaware of the clinical signs of lameness.

**Table-5:** Dairy farmers (n = 155) knowledge of the clinical sign of lameness.

Questions	M1 (%)		M2 (%)		M3 (%)		Total (%)		Chi- square	p-value
			
	Yes	No	Yes	No	Yes	No	Yes	No		
Movement problem	38 (24.5)	4 (2.6)	62 (40)	2 (1.3)	46 (29.7)	3 (2)	**146 (94.2)**	9 (5.8)	3.8	0.147
Decreases appetite	35 (22.6)	7 (4.5)	62 (40)	1 (0.6)	48 (30.9)	2 (1.3)	145 (93.5)	10 (6.5)	10.6	0.005
Reduces milk yield	36 (23.2)	6 (3.9)	61 (39.4)	2 (1.3)	49 (31.6)	1 (0.6)	146 (94.2)	9 (5.8)	8.2	0.017
Loss of mobility	38 (24.5)	4 (2.6)	61 (39.4)	2 (1.3)	44 (28.4)	6 (3.9)	**143 (92.3)**	12 (7.7)	3.3	0.190
Foot rot	39 (25.1)	2 (1.3)	61 (39.4)	2 (1.3)	48 (30.9)	3 (2)	**148 (95.5)**	7 (4.5)	0.5	0.780
Increase lying times	36 (23.2)	5 (3.2)	62 (40)	1 (0.6)	42 (27.1)	9 (5.8)	140 (90.3)	15 (9.7)	8.7	0.013
Reduces reproductive ability	37 (23.9)	4 (2.6)	59 (38.1)	4 (2.6)	47 (30.3)	4 (2.6)	**143 (92.3)**	12 (7.7)	0.6	0.748
Reduce body weight	37 (23.9)	3 (2)	60 (38.7)	3 (2)	49 (31.6)	3 (2)	**146 (94.2)**	9 (5.8)	0.6	0.732
							*Pass mark 4.69 (58.56%)			

* Statistically significant at P ≤ 0.05, M1=uMgungundlovu, M2=uMpofana, M3=uMngeni, (Correct answers are highlighted in bold. *pass mark has been computed by the addition of percentages in which most of respondents correctly answered the question and divided by 100)

The majority of the respondents (91%) acknowledged that lameness should be treated by applying a block to the unaffected claw (Table-6). However, others believed that treating lame cows with Savlon (Imperial Chemical Industries, UK) was critical (88%). The correlation between farm localities, application of the block to unaffected claws, and treatment with Savlon (Imperial Chemical Industries) was significant (p < 0.05).

**Table-6:** Dairy farmworkers (n = 155) knowledge of treatment of lameness.

Questions	M1 (%)		M2 (%)		M3 (%)		Total		Chi- square	p-value
	Yes	No	Yes	No	Yes	No	Yes	No		
Trimming regular	33 (21.3)	8 (5.2)	60 (38.7)	3 (2)	45 (29)	6 (3.9)	138 (89)	17 (11)	5.6	0.061
Apply bandage on the affected claw	33 (21.3)	7 (4.5)	62 (40)	2 (1.3)	46 (29.7)	5 (3.2)	141 (90.9)	14 (9.1)	8.2	0.017
Apply block on unaffected claw	37 (23.9)	5 (3.2)	52 (33.5)	10 (6.5)	39 (25.2)	12 (7.7)	128 (82.6)	27 (17.4)	1.6	0.451
Foot bath	39 (25.2)	2 (1.3)	61 (39.3)	2 (1.3)	47 (30.3)	4 (2.6)	147 (94.8)	8 (5.2)	1.3	0.532
Antibiotics	36 (23.2)	5 (3.2)	61 (39.3)	2 (0.3)	49 (31.6)	2 (1.3)	146 (94.2)	9 (5.8)	4.2	0.123
Treating with savlon	30 (19.4)	11 (7.1)	58 (37.4)	5 (3.2)	49 (31.6)	2 (1.6)	137 (88.4)	18 (11.6)	13.0	0.001
Trim and put a plank in severely trimmed leg	33 (21.3)	8 (5.2)	58 (37.4)	5 (3.2)	47 (30.3)	4 (2.6	138 (89)	17 (11)	4.2	0.124

*Statistically significant p ≤ 0.05, M1=uMgungundlovu, M2=uMpofana, M3=uMngeni

Several respondents (96%) in this study (Table-7) believe that not allowing animals to travel long distances can prevent lameness. The interaction between prevention and farm localities was significant (p < 0.05). While other respondents agreed on trimming hooves twice a year (90%) and scrubbing manure in the feed pad (91%), they disagreed on scrubbing manure in the feed pad to avoid infectious diseases. All these variables were significant at (p < 0.05). Furthermore, majority of respondents believed that all concrete should be grooved to prevent cows from slipping (89%), and the variance was significant (p < 0.05).

**Table-7:** Dairy farmers (n = 155) knowledge of the prevention and management of lameness.

Questions	M1 (%)		M2 (%)		M3 (%)		Total		Chi- square	p-value
			
	Yes	No	Yes	No	Yes	No	Yes	No		
Do not allow animals to travel long distance	37 (23.9)	4 (2.6)	63 (40.6)	0	48 (30.9)	3 (2)	148 (95.4)	7 (4.6)	5.8	0.055
Do not allow animals to mix with contaminated animals	35 (22.6)	6 (3.9)	61 (39.4)	2 (1.3)	48 (30.9)	3 (2)	144 (92.9)	11 (7.1)	5.1	0.077
Spray infected pastures	36 (23.2)	5 (3.2)	54 (34.8)	9 (5.8)	41 (26.5)	10 (6.5)	131 (84.5)	24 (15.5)	1.1	0.585
Trimming hoofs twice a year	32 (20.6)	9 (5.8)	58 (37.4)	5 (3.2)	49 (31.6)	2 (1.3)	139 (89.7)	16 (10.3)	8.6	0.013
Early identification of lame cows	36 (23.2)	5 (3.2)	58 (37.4)	5 (3.2)	46 (29.7)	5 (3.2)	140 (90.3)	15 (9.7)	0.5	0.772
Feeding proper nutrition for thin (Poor BCS) animals	32 (20.6)	9 (5.8)	57 (36.8)	6 (3.9)	46 (29.7)	5 (3.2)	135 (87.1)	20 (12.9)	4.1	0.131
Scrub the manure in the feed pad to avoid infectious diseases	34 (21.9)	7 (4.5)	57 (36.8)	6 (3.9)	50 (32.3)	1 (0.6)	141 (91)	14 (9)	6.3	0.042
Apply footbath every week	36 (23.2)	5 (3.2)	59 (38.1)	4 (2.6)	49 (31.6)	2 (1.3)	144 (92.9)	11 (7.1)	2.5	0.294
All concrete should be grooved to prevent cows from slipping	32 (20.6)	9 (5.8)	58 (37.4)	5 (3.2)	48 (31)	3 (2)	138 (89)	17 (11)	7.0	0.030

*Statistically significant at p ≤ 0.05, M1=uMgungundlovu, M2=uMpofana, M3=uMngeni

The movement problem was related to age, gender, and occupation level (p < 0.05) (Table-8). However, demographic information (ethnicity, marital status, and occupation level) was significant (p < 0.05) when associated with decreased appetite. Furthermore, foot rot and increased lying time were correlated with age and occupation level (p < 0.05).

**Table-8:** Association between demographic information and dairy farmworkers (n = 155) knowledge in clinical signs of lameness.

Demography	Movement problem	Decrease appetite	Reduce milk yield	Loss mobility	Foot rot	Increase lying time	Reduce reproduction	Reduce body weight
Age	0.001*	0.003*	0.282	0.511	0.016*	0.030*	0.020*	0.683
Gender	0.048*	0.307	0.545	0.907	0.330	0.703	0.124	0.725
Ethnic	0.515	0.032*	0.128	0.361	0.476	0.526	0.284	0.255
Marital status	0.709	0.012*	0.013*	0.710	0.552	0.499	0.892	0.814
Experience	0.107	0.927	0.596	0.110	0.180	0.110	0.501	0.468
Education level	0.375	0.949	0.592	0.013*	0.236	0.219	0.240	0.500
Occupation	0.050*	0.001*	0.763	0.929	0.052*	0.045*	0.864	0.709

* Statistically significant (p ≤ 0.05)

## Discussion

The current study revealed that 63.9% of respondents were males than females (36.1%). This could be because most of the work done in dairy farming requires men’s power, such as hoof trimming and other farm practices. The results are similar to those reported in the Eastern Cape Province [37]. The current study revealed that 83.2% of the respondents were aged between 21 and 40. In the dairy industry and among the energetic group, this could be the most desirable age. The findings align with reports from the Eastern Cape Province, South Africa [37], Indonesia [14], and Spain [38] emphasizing youth and energy as mandatory dairy farming components.

Majority of the respondents employed pasture-based dairy systems, which were linked to lameness incidents through their use and milking parlor types. Cow constant walking on uneven terrain and long distances to access grazing areas and water sources predispose the foot to lameness [39–41]. Cattle hooves are prone to lameness due to walking. These findings harmonize with U.S reports indicating that environmental factors and the style of management system can add to the likelihood of lameness [20].

About 81% of the respondents had knowledge about bovine lameness, while 19% did not. Farm training and awareness campaigns about lameness and mastitis may result in the culling of dairy cattle. Our findings correspond with those from the Eastern Cape Province [37]. Among farmers and producers in the study population, there is a satisfactory grasp of the disease. About 81% of the respondents grasped the concept of lameness as outlined in the study (Table-3). Findings in dairy animals with claw or limb wounds indicate similarities to reports from Europe [3, 16, 42]. Numerous studies confirm the frequent cause of lameness is claw wounds [1, 41, 43, 44]. The underlying pathogenesis of chronic wounds may also cause different etiologies of wounds to be affected by bacteria in different ways [45]. Deep digit infections, like sole or hoof wall wounds, often stem from injuries. The swelling of soft tissues near the coronary band is a common outcome of these injuries [44, 46, 47].

In summer, the majority of respondents reported a higher incidence of lameness compared to other seasons. Summer rainfall can result in wetness and mud. Dairy cows may experience more hoof injuries and bacterial infections [12]; respondents generally believed that untrimmed cattle have a higher risk for lameness than their trimmed counterparts. The retention of dirt and pathogens in untrimmed hair and hooves may be a cause [8, 13, 38]. The findings in this study correspond with those in Ethiopia [10], Europe [16], and South America [48]. Our findings coincide with previous study conducted in Australia [49].

The respondents of the current study agreed that the Friesland is a breed prone to lameness. The Netherlands [11, 50] reported similar results. The Friesland breed, known for its large udder and long hoof, is significantly more susceptible to lameness than other breeds, according to numerous studies [9, 23, 27, 51, 52]. Crossbreeds also suffer from lameness due to the presence of the Friesland gene [38, 53–56].

According to this study, increasing parity increases the likelihood of lameness in dairy cows. As a result, these findings are similar to those reported in Canada, where lameness prevalence increased with increasing parity; compared with the first parity, cows in parity 2, 3, and 4 had a higher prevalence of lameness [29, 34, 57]. Because older cows produce more milk than the first lactating cows, there is a greater chance of lameness developing as the cow ages. Other researchers have found that cows in the third to fourth parity are more likely to become lame in Finland [58].

Most respondents in the current study recognized lameness symptoms. About 60%–71% was the average respondent’s level of knowledge on clinical signs of lameness, and their pass rate was 5.6 out of 8. The European findings validate that many farm workers comprehend cows’ lameness symptoms [3, 16]. Among farm workers, the movement problem is a well-known clinical sign of lameness [4, 59]. Daily observation of cows entering and exiting the milking parlor allows farm workers to identify movement problems.

Lame cows consume less feed because they spend so much time lying down, which lowers their milk production [60, 61]. Our findings align exactly with the study conducted in New York [8, 20, 43]. According to other investigations, lameness is the primary reason for decreased milk production [30, 38, 62]. Milk reduction is an indicator of lameness, based on previous studies [63]. Decreased mobility may reduce feed intake and, ultimately, milk production. Studies show a correlation between reduced feed intake and decreased milk production [17, 56, 62, 64].

The majority of respondents agreed that bandaging the affected claw was the best way to treat lameness. These findings are comparable to those reported in Europe [3], Asia [40], Mexico [65], and Malaysia [66]. However, in some studies, bandages should be removed within 24 h because it can cause infection in the claw [4, 13, 43, 67]. At the same time, some claim that another treatment for lame cows is the application of Savlon (Imperial Chemical Industries) in the affected claw [30, 31, 68]. Salvon (Imperial Chemical Industries) will not reduce milk yield, and no antibiotics will be injected into affected lame cows.

The prevention and management of lameness in dairy cows are critical for avoiding premature culling of lame cows [7, 30, 69]. It is stated that dairy cows are not permitted to travel long distances because doing so will damage their hoofs and reduce milk yield. Furthermore, hoof trimming is recommended twice a year by a hoof care center or a veterinarian [18, 29, 70]. These findings are consistent with those reported in Asia [40], the Netherlands [11], and the US [33]. Hoof trimming reduces the likelihood of the herd becoming lame. Bovine lameness prevention necessitates a multifaceted approach that includes proper nutrition, regular hoof trimming, clean and dry living conditions, footbaths, minimizing standing time, and genetics [3, 50, 71]. Dairy farmers can reduce the risk of lameness and improve their herds’ overall health and welfare by implementing these strategies [40, 60, 72].

## Conclusion

This study revealed no correlation between an individual’s level of education and their understanding or application of lameness to boost productivity. Despite having access to information about 17 risk factors for lameness, farm workers could only identify four: summer, parity, untrimmed hooves, and wounds on lower limbs. This emphasizes their lack of understanding of lameness. In the dairy industry, training workers to enhance their knowledge is necessary. Effective management and mitigation of bovine lameness require ongoing training and retraining of farm workers.

## Data Availability

The supplementary data can be available from the corresponding author on a request.

## Authors’ Contributions

SM and IFJ: Conceptualization and review and editing of the manuscript. SM: Writing–original draft preparation and data curation. All authors read and approved the final manuscript.

## References

[1] Griffiths B.E, White D.G, Oikonomou G (2018). A cross-sectional study into the prevalence of dairy cattle lameness and associated herd-level risk factors in England and Wales. Front. Vet. Sci.

[2] Mas-Coma S, Bargues M.D, Valero M.A (2005). Fascioliasis and other plant-borne trematode zoonoses. Int. J. Parasitol.

[3] Wensman J, Marcia A, Guimarães S, Garvey M (2022). Lameness in dairy cow herds:Disease aetiology, prevention and management. Dairy.

[4] Heinonen M, Peltoniemi O, Valros A (2013). Impact of lameness and claw lesions in sows on welfare, health and production. Livest. Sci.

[5] Elliott T.P, Kelley J.M, Rawlin G, Spithill T.W (2015). High prevalence of fasciolosis and evaluation of drug efficacy against Fasciola hepatica in dairy cattle in the Maffra and Bairnsdale districts of Gippsland, Victoria, Australia. Vet. Parasitol.

[6] Weber A, Stamer E, Junge W, Thaller G (2013). Genetic parameters for lameness and claw and leg diseases in dairy cows. J. Dairy Sci.

[7] Sadiq M.B, Ramanoon S.Z, Shaik Mossadeq W.M.M, Mansor R, Syed-Hussain S.S (2021). Preventive hoof trimming and animal-based welfare measures influence the time to first lameness event and hoof lesion prevalence in dairy cows. Front. Vet. Sci.

[8] Warnick L.D, Janssen D, Guard C.L, Gröhn Y.T (2001). The effect of lameness on milk production in dairy cows. J. Dairy Sci.

[9] Antanaitis R, Juozaitienė V, Urbonavičius G, Malašauskienė D, Televičius M, Urbutis M, Baumgartner W (2021). Impact of lameness on attributes of feeding registered with noseband sensor in fresh dairy cows. Agriculture (Switzerland).

[10] Sheferaw D, Abebe R, Megersa B, Amenu K, Abunna F, Regassa A, Denbarga Y, Fekadu A, Mekibib B, Rebuma E, Abera E, Sefiw G, Hordofa D, Ashebo A, Wako F (2021). Dairy cattle lameness prevalence, causes and risk factors in selected farms of southern Ethiopia. Ethiop. Vet. J.

[11] Van der Linde C, De Jong C, Koenen E.P.C, Eding H (2010). Claw health index for Dutch dairy cattle based on claw trimming and conformation data. J. Dairy Sci.

[12] Randall L.V, Green M.J, Chagunda M.G.G, Mason C, Archer S.C, Green L.E, Huxley J.N (2015). Low body condition predisposes cattle to lameness:An 8-year study of one dairy herd. J. Dairy Sci.

[13] Charfeddine N, Pérez-Cabal M.A (2017). Effect of claw disorders on milk production, fertility, and longevity, and their economic impact in Spanish Holstein cows. J. Dairy Sci.

[14] Villa-Mancera A, Reynoso-Palomar A (2019). High prevalence, potential economic impact, and risk factors of Fasciola hepatica in dairy herds in tropical, dry and temperate climate regions in Mexico. Acta Trop.

[15] Gelasakis A.I, Kalogianni A.I, Bossis I (2019). Aetiology, risk factors, diagnosis and control of foot-related lameness in dairy sheep. Animals (Basel).

[16] Nalon E, Stevenson P (2019). Addressing lameness in farmed animals:An urgent need to achieve compliance with EU animal welfare law. Animals (Basel).

[17] Bruijnis M.R.N, Hogeveen H, Garforth C.J, Stassen E.N (2013). Dairy farmers'attitudes and intentions towards improving dairy cow foot health. Livest. Sci.

[18] Langova L, Novotna I, Nemcova P, Machacek M, Havlicek Z, Zemanova M, Chrast V (2020). Impact of nutrients on the hoof health in cattle. Animals (Basel).

[19] Arnott G, Ferris C.P, O'connell N.E (2017). Review:Welfare of dairy cows in continuously housed and pasture-based production systems. Animal.

[20] Wynands E.M, Roche S.M, Cramer G, Ventura B.A (2021). Dairy farmer, hoof trimmer, and veterinarian perceptions of barriers and roles in lameness management. J. Dairy Sci.

[21] Ózsvári L (2017). Economic cost of lameness in dairy cattle herds. J. Dairy Vet. Anim. Res.

[22] Green L.E, Hedges V.J, Schukken Y.H, Blowey R.W, Packington A.J (2002). The impact of clinical lameness on the milk yield of dairy cows. J. Dairy Sci.

[23] Nalon E, Conte S, Maes D, Tuyttens F.A.M, Devillers N (2013). Assessment of lameness and claw lesions in sows. Livest. Sci.

[24] Holzhauer M, Hardenberg C, Bartels C.J.M, Frankena K (2006). Herd-and cow-level prevalence of digital dermatitis in the Netherlands and associated risk factors. J. Dairy Sci.

[25] Sanders A.H, Shearer J.K, De Vries A (2009). Seasonal incidence of lameness and risk factors associated with thin soles, white line disease, ulcers, and sole punctures in dairy cattle. J. Dairy Sci.

[26] Buttchereit N, Stamer E, Junge W, Thaller G (2012). Genetic parameters for energy balance, fat/protein ratio, body condition score and disease traits in German Holstein cows. J. Anim. Breed. Genet.

[27] Häggman J, Juga J (2013). Genetic parameters for hoof disorders and feet and leg conformation traits in Finnish Holstein cows. J. Dairy Sci.

[28] Njoroge S.M, Musotsi P.Y, Otieno C.A, Njoroge M (2017). Prevalence of fasciolosis in cattle, sheep, and goats slaughtered in Slaughter Slabs in Trans-Nzoia West, Kenya. and knowledge of livestock handlers. J. Biol. Agric. Healthc.

[29] Cutler J.H.H, Rushen J, De Passillé A.M, Gibbons J, Orsel K, Pajor E, Barkema H.W, Solano L, Pellerin D, Haley D, Vasseur E (2017). Producer estimates of prevalence and perceived importance of lameness in dairy herds with tiestalls, freestalls, and automated milking systems. J. Dairy Sci.

[30] Wilson J.P, Randall L.V, Green M.J, Rutland C.S, Bradley C.R, Ferguson H.J, Bagnall A, Huxley J.N (2021). A history of lameness and low body condition score is associated with reduced digital cushion volume, measured by magnetic resonance imaging, in dairy cattle. J. Dairy Sci.

[31] Davis-Unger J, Schwartzkopf-Genswein K.S.J, Pajor E.A, Hendrick S, Marti S, Dorin C, Orsel K (2019). Prevalence and lameness-associated risk factors in Alberta feedlot cattle. Transl. Anim. Sci.

[32] Pritchard T, Coffey M, Mrode R, Wall E (2013). Understanding the genetics of survival in dairy cows. J. Dairy Sci.

[33] Salfer J.A, Siewert J.M, Endres M.I (2018). Housing, management characteristics, and factors associated with lameness, hock lesion, and hygiene of lactating dairy cattle on Upper Midwest United States dairy farms using automatic milking systems. J. Dairy Sci.

[34] Matson R.D, King M.T.M, Duffield TF, Santschi D.E, Orsel K, Pajor E.A, Penner G.B, Mutsvangwa T, DeVries T.J (2022). Farm-level factors associated with lameness prevalence, productivity, and milk quality in farms with automated milking systems. J. Dairy Sci.

[35] Blackie N, Amory J.R, Bleach E, Scaife J.R (2011). The effect of lameness on lying behaviour of zero grazed Holstein dairy cattle. Appl. Anim. Behav. Sci.

[36] Browne N, Hudson C.D, Crossley R.E, Sugrue K, Kennedy E, Huxley N, Conneely M (2022). Lameness prevalence and management practices on Irish pasture-based dairy farms. Ir. Vet. J.

[37] Diniso Y.S, Jaja I.F (2021). A retrospective survey of the factors responsible for culling and mortality in dairy farms in the Eastern Cape Province, South Africa. Sci. Afr.

[38] Pérez-Cabal M.A, Alenda R (2014). Clinical lameness and risk factors in a Spanish Holstein population. Livest. Sci.

[39] Kang X, Zhang X.D, Liu G (2020). Accurate detection of lameness in dairy cattle with computer vision:A new and individualized detection strategy based on the analysis of the supporting phase. J. Dairy Sci.

[40] Solano L, Barkema H.W, Pajor E.A, Mason S, LeBlanc S.J, Zaffino Heyerhoff J.C, Nash C.G.R, Haley D.B, Vasseur E, Pellerin D, Rushen J, De Passillé A.M, Orsel K (2015). Prevalence of lameness and associated risk factors in Canadian Holstein-Friesian cows housed in freestall barns. J. Dairy Sci.

[41] Hund A, Chiozza Logroño J, Ollhoff R.D, Kofler J (2019). Aspects of lameness in pasture based dairy systems. Vet. J.

[42] FVE (Federation of Veterinarians of Europe) (2019). FVE Position on Welfare and Dairy Cows:Lameness.

[43] Foditsch V, Oikonomou G, Machado V.S, Bicalho M.L, Ganda E.K, Lima S.F, Rossi R, Ribeiro B.L, Kussler A, Bicalho R.C (2016). Lameness prevalence and risk factors in large dairy farms in upstate New York. Model development for the prediction of claw horn disruption lesions. PLoS One.

[44] Knage-Rasmussen K.M, Houe H, Rousing T, Sorensen J.T (2014). Herd-and sow-related risk factors for lameness in organic and conventional sow herds. Animal.

[45] Sogstad A.M, Østerås O, Fjeldaas T (2006). Bovine claw and limb disorders related to reproductive performance and production diseases. J. Dairy Sci.

[46] Han G, Ceilley R (2017). Chronic wound healing:A review of current management and treatments. Adv. Ther.

[47] Puca V, Marulli R.Z, Grande R, Vitale I, Niro A, Molinaro G, Prezioso S, Muraro R, Di Giovanni P (2021). Microbial species isolated from infected wounds and antimicrobial resistance analysis:Data emerging from a three-years retrospective study. Antibiotics (Basel).

[48] Vargas-Bello-Pérez E, Miranda-de la Lama G.C, Teixeira D.L, Enríquez-Hidalgo D, Tadich T, Lensink J (2017). Farm animal welfare influences on markets and consumer attitudes in Latin America:The cases of Mexico, Chile and Brazil. J. Agric. Environ. Ethics.

[49] Stott K.J, Gourley C.J.P (2016). Intensification, nitrogen use and recovery in grazing-based dairy systems. Agric. Syst.

[50] Somers J.G.C.J, Frankena K, Noordhuizen-Stassen E.N, Metz J.H.M (2005). Risk factors for digital dermatitis in dairy cows kept in cubicle houses in The Netherlands. Prev. Vet. Med.

[51] Alawneh J.I, Laven R.A, Stevenson M.A (2011). The effect of lameness on the fertility of dairy cattle in a seasonally breeding pasture-based system. J. Dairy Sci.

[52] Jaja I.F, Mushonga B, Green E, Muchenje V (2017). Seasonal prevalence, body condition score and risk factors of bovine fasciolosis in South Africa. Vet. Anim. Sci.

[53] Supakorn C, Stock J.D, Garay E, Johnson A.K, Stalder K.J (2018). Lameness:A principle problem to sow longevity in breeding herds. CAB Rev.

[54] Koenig S, Sharifi A.R, Wentrot H, Landmann D, Eise M, Simianer H (2005). Genetic parameters of claw and foot disorders estimated with logistic models. J. Dairy Sci.

[55] Barker Z.E, Leach K.A, Whay H.R, Bell N.J, Main D.C.J (2010). Assessment of lameness prevalence and associated risk factors in dairy herds in England and Wales. J. Dairy Sci.

[56] Buch L.H, Sørensen A.C, Lassen J, Berg P, Eriksson J.T, Jakobsen J.H, Sørensen M.K (2011). Hygiene-related and feed-related hoof diseases show different patterns of genetic correlations to clinical mastitis and female fertility. J. Dairy Sci.

[57] Jewell M.T, Cameron M, Spears J, McKenna S.L, Cockram M.S, Sanchez J, Keefe G.P (2019). Prevalence of lameness and associated risk factors on dairy farms in the Maritime Provinces of Canada. J. Dairy Sci.

[58] Sarjokari K, Kaustell K.O, Hurme T, Kivinen T, Peltoniemi O.A.T, Saloniemi H, Rajala-Schultz P.J (2013). Prevalence and risk factors for lameness in insulated free stall barns in Finland. Livest. Sci.

[59] Browne N, Hudson C.D, Crossley R.E, Sugrue K, Kennedy E, Huxley J.N, Conneely M (2022). Cow-and herd-level risk factors for lameness in partly housed pasture-based dairy cows. J. Dairy Sci.

[60] Beaver A, Weary D.M, Von Keyserlingk M.A.G (2013). Invited review:The welfare of dairy cattle housed in tiestalls compared to less-restrictive housing types:A systematic review. J. Dairy Sci.

[61] Sadiq M.B, Ramanoon S.Z, Mossadeq W.M.S, Mansor R, Syed-Hussain S.S (2017). Association between lameness and indicators of dairy cow welfare based on locomotion scoring, body and hock condition, leg hygiene and lying behavior. Animals (Basel).

[62] Daros R.R, Eriksson H.K, Weary D.M, Von Keyserlingk M.A.G (2020). The relationship between transition period diseases and lameness, feeding time, and body condition during the dry period. J. Dairy Sci.

[63] King M.T.M, LeBlanc S.J, Pajor E.A, DeVries T.J (2017). Cow-level associations of lameness, behavior, and milk yield of cows milked in automated systems. J. Dairy Sci.

[64] Barker Z.E, Vázquez Diosdado J.A, Codling E.A, Bell N.J, Hodges H.R, Croft D.P, Amory J.R (2018). Use of novel sensors combining local positioning and acceleration to measure feeding behavior differences associated with lameness in dairy cattle. J. Dairy Sci.

[65] Randall L.V, Green M.J, Green L E, Chagunda M.G.G, Mason C, Archer S.C, Huxley J.N (2018). The contribution of previous lameness events and body condition score to the occurrence of lameness in dairy herds:A study of 2 herds. J. Dairy Sci.

[66] Sadiq M.B, Ramanoon S.Z, Mansor R, Syed-Hussain S.S, Shaik Mossadeq W.M (2017). Prevalence of lameness, claw lesions, and associated risk factors in dairy farms in Selangor, Malaysia. Trop. Anim. Health Prod.

[67] Anyanwu M.U, Jaja I.F, Okpala C.O.R, Jaja C.J.I, Oguttu J.W, Chah K.F, Shoyinka V.S (2021). Potential sources and characteristic occurrence of mobile colistin resistance (mcr) gene-harbouring bacteria recovered from the poultry sector:A literature synthesis specific to high-income countries. PeerJ.

[68] Whay H (2002). Locomotion scoring and lameness detection in diary cattle. Practice.

[69] Van Nuffel A, Zwertvaegher I, Pluym L, Van Weyenberg S, Thorup V.M, Pastell M, Sonck B, Saeys W (2015). Lameness detection in dairy cows:Part 1. How to distinguish between non-lame and lame cows based on differences in locomotion or behavior. Animals (Basel).

[70] Arias-Pacheco C, Lucas J.R, Rodríguez A, Córdoba D, Lux-Hoppe E.G (2020). Economic impact of the liver condemnation of cattle infected with Fasciola hepatica in the Peruvian Andes. Trop. Anim. Health Prod.

[71] Lawrence K, Chesterton R, Laven R (2011). Further investigation of lameness in cows at pasture:An analysis of the lesions found in, and some possible risk factors associated with, lame New Zealand dairy cattle requiring veterinary treatment. J. Dairy Sci.

[72] Christodoulopoulos G (2009). Foot lameness in dairy goats. Res. Vet. Sci.

